# Radiolabeling of Cramoll 1,4: Evaluation of the Biodistribution

**DOI:** 10.1155/2011/945397

**Published:** 2011-06-30

**Authors:** Beatriz Ferreira de Carvalho Patricio, Maria Helena Madruga Lima-Ribeiro, Maria Tereza dos Santos Correia, Ana Maria dos Anjos Carneiro-Leão, Marta de Souza Albernaz, Thiago Barboza, Sergio Augusto Lopes de Souza, Ralph Santos-Oliveira

**Affiliations:** ^1^Laboratory of Nanoradiopharmaceuticals, Academical Hospital Clementino Fraga Filho, 21941-913 Rio de Janeiro, RJ, Brazil; ^2^Department of Morphological and Biochemical Analysis, Rural University of Pernambuco, 52171-900 Recife, PE, Brazil; ^3^Department of Biochemistry, Federal University of Pernambuco, 52171-900 Recife, PE, Brazil; ^4^Departamento de Radiologia, Hospital Universitário Clementino Fraga Filho Laboratório de Marcação de Células e Moléculas, Universidade Federal do Rio de Janeiro, 21941-901 Rio de Janeiro, RJ, Brazil

## Abstract

The cramoll 1,4 is a well-studied lectin. However, few studies about its biodistribution have been done before. In this study, we radiolabeled the cramol 1,4 with Tc-99m and analyzed the biodistribution. The results showed that the cramol has an abnormal uptake by the bowel with reflections on its clearance mechanism.

## 1. Introduction

Lectins are proteins frequently found in cellular surfaces or in intracellular particles [1]. They possess binding sites specific to carbohydrates and have the capacity to interact with molecules of biological fluids and those present in cellular surfaces [2]. Lectins can substitute natural linkers and activate cellular responses through different roads of intracellular signalling or endocytosis of formed compounds [3, 4]. The therapeutic use of lectins in wound healing process is still scarcely studied.

Camaratu or camaratuba bean (*Cratylia mollis*) is forage of the Northeast Semi-Arid Region from Brazil. Molecular forms of *C. mollis* seed lectin (Cramoll 1, Cramoll 2, Cramoll 3, and Cramoll 4) have been highly purified and characterized (de Souza et al., 2003) [2, 5]. Cramoll 1, Cramoll 2, and Cramoll 4 specifically bind glucose/mannose, while Cramoll 3 is a galactose-specific glycoprotein. Preparations containing isoforms (Cramoll 1,4) and isolectins (Cramoll 1,2,3), as well as Cramoll 1, have been studied in structural analyses and for the most several biotechnological applications (de Souza et al., 2003) [6–12]. 

The use of nuclear technique for the evaluation of biomolecules and medicines although old is not widely used even nowadays. Since the discovery of the element Technetium, in 1947, it has been used for many purposes. Among the various uses, the labeling process for molecules is the most well established. Scintigraphy imaging provides a dimensional method for exactly locating gamma emitters in a noninvasive procedure under in vivo conditions. For the characterization of drugs, and biomolecules, molecular imaging techniques are extremely helpful to follow biodistribution in experimental animal studies [13]. In this study, the isolectins Cramol 1,4 were radiolabeled with Tc-99m for the evaluation of the biodistribution and in order to forecast a possible use as radiopharmaceutical in healing process.

## 2. Methods

### 2.1. Chromatography

The labeling processes were made using 150 *μ*L of cramol (isoform 1,4) solution incubated with stannous chloride (SnCl_2_) solutions (80 *μ*L/mL) (Sigma-Aldrich) for 20 minutes at room temperature. Then, this solution was incubated with 100 *μ*Ci (approximately 300 *μ*L) of technetium-99m (IPEN/CNEN) for other 10 minutes in order to label the cramol 1,4 (here just called cramol) with Tc-99m.

In order to characterize the labeled cramol, thin-layer chromatography (TLC) was made using paper Whatman no. 1. The TLC was performed using 2 *μ*L of a labeled sample in acetone (Proquimios) as a mobile phase. The radioactivity of the strips was verified in a gamma counter (Packard, Cobra II) as described in Table 1.

### 2.2. Biodistribution

**Figure 1:**
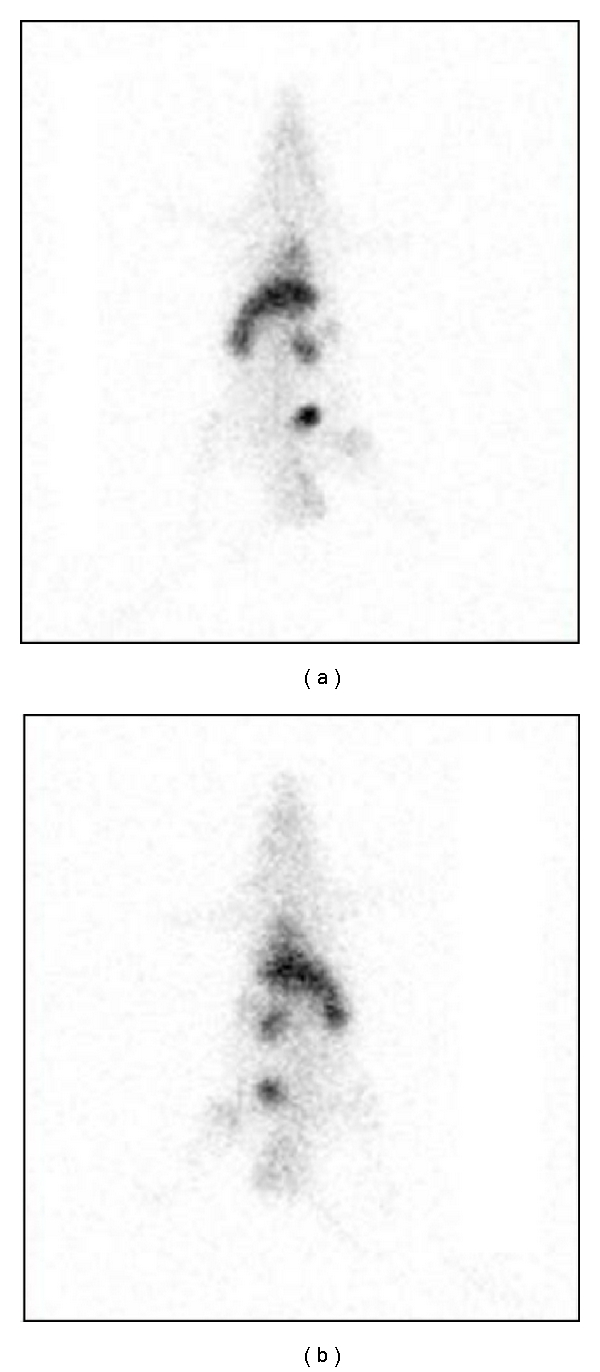
Biodistribution of cramol in rats.

**Figure 2:**
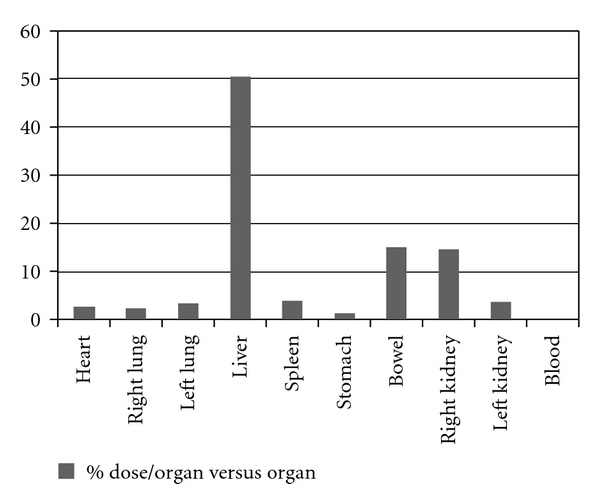
Biodistribution %dose/organ versus organ.

**Figure 3:**
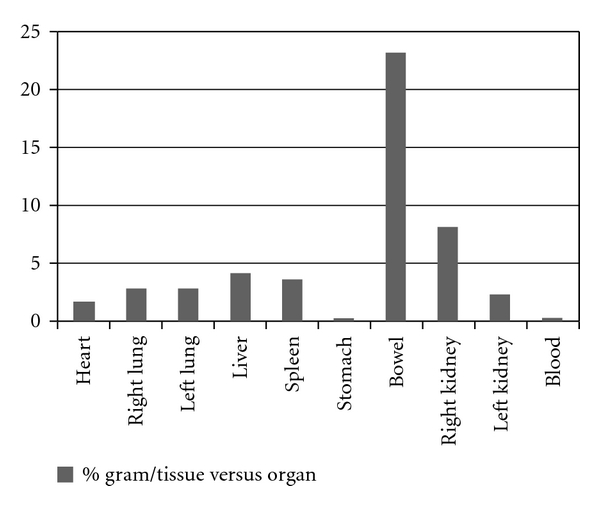
Biodistribution %gram/tissue versus organ.

The biodistribution was made using one male Wistar rat. In this direction, the Institutional Review Board and the Animal Ethics Committee approved the study protocol for this study. The labeled samples (3,7 MBq/0,2 mL) were administered after the catheterization of the jugular vein. Planar images were obtained 1 hour after injection at a Millennium Gamma Camera (GE Healthcare, Cleveland, USA). Counts were acquired for 5 min in a 15% window centered at 140 KeV (Figure 1). Then, animals were sacrificed, their were organs removed and weighted, and the radioactivity uptake was counted in a gamma counter (Packard-Cobra II). Results were expressed as the percentage of injected dose per organ and percent of injected dose per gram of tissue (Table 2, Figures 2 and 3).

## 3. Results and Discussion

### 3.1. Whatman no. 1 Chromatography

Results are shown in Table 1.

In this case, the results showed that the acetone can be used for this purpose. In a triplicate test, all the results were very close. The free pertechnetate was eluted to the top of the chromatograms, and the cramol stayed in the bottom. Moreover, the results observed in the chromatogram elucidated the question about the efficacy of the labeling process, corroborating that the cramol was efficiently labeled with Tc-99m.

### 3.2. Biodistribution Studies

The results of the labeled sample are as shown below.

^99m^Tc-Cramol scintigraphy 1 h after injection shows liver, kidneys, and bladder. A: anterior view. B: posterior view. 

As we can see, the great part of the lectin was processed by the liver. This is absolutely normal especially for injectable drugs, since almost all the drugs are first processed by the hepatic system (Figure 2). Otherwise, when dissecting, weighting and counting the organs, a suspicious data appeared.  Figure 3 showed an abnormal accumulation of the lectin in the bowel. As a protein, we were expecting that its clearance would be made by the kidney, and in fact, it occurred. However, the rate between the bowel and the kidney is over the double. In this case, the clearance time would be reduced and the reabsorption of the drug could be facilitated by the microvilli of the bowel. In this case a dosage error could occur or even a toxic reaction due its accumulation in the bowel. In both cases, further studies should be made to clarify this mechanism and the relevance of that information in a developed drug based on lectin similar to the cramol 1,4. It is interesting to notice, analyzing both Figures 1 and 2, that the lectin cannot pass the hematoencephalic barrier. And in this case, the lectin does not represent a risk for the brain function.

## 4. Conclusion

The radiolabeling of the cramol showed that this lectin has an uncommon behavior specially related to the clearance mechanism that we believe must be more studied. It also showed that the nuclear technique can be very helpful for the development of drugs and medicines. The results help to elucidate that the lectin cannot cross the hematoencephalic barrier.

## Figures and Tables

**Table 1:** Ascending chromatographs of the ^99m^Tc-cramol comparing to free pertechnetate (Na^99m^TcO_4_^−^).

	Solvent	Bottom (%)	Top (%)
^ 99m^Tc-cramol	Acetone	89.4	10.6
Na^99m^TcO_4_^−^	Acetone	0.3	99.7

**Table 2:** Biodistribution of the labeled samples in rat.

Organs	%dose/organ	%gram/tissue
Heart	2,98	1,78
Right lung	2,65	2,93
Left lung	3,58	2,88
Liver	50,82	4,17
Spleen	4,28	3,69
Stomach	1,44	0,38
Intestine	15,38	23,30
Right kidney	14,83	8,23
Left kidney	3,77	2,35
Blood	0,27	0,27
